# Advances in the Remote Sensing of Terrestrial Evaporation

**DOI:** 10.3390/rs11091138

**Published:** 2019-05-13

**Authors:** Matthew F McCabe, Diego Miralles, Thomas R.H. Holmes, Joshua B Fisher

**Affiliations:** 1 Water Desalination and Reuse Center, Division of Biological and Environmental Sciences and Engineering, King Abdullah University of Science and Technology, Thuwal, Saudi Arabia; 2 Laboratory of Hydrology and Water Management, Ghent University, Coupure Links 653, 9000 Ghent, Belgium; 3 Hydrological Sciences Laboratory, NASA Goddard Space Flight Center, Greenbelt, MD 20771, USA; 4 Jet Propulsion Laboratory, California Institute of Technology, Pasadena, CA 91109, USA

**Keywords:** evaporation, remote sensing, Earth observation, novel sensing, land surface modeling, land surface flux, cubesats, high-resolution

## Abstract

Characterizing the terrestrial carbon, water and energy cycles depends strongly on a capacity to accurately reproduce the spatial and temporal dynamics of land surface evaporation. For this, and many other reasons, monitoring terrestrial evaporation across multiple space and time scales has been an area of focused research for many decades. Much of this activity has been supported by developments in satellite remote sensing, which have been leveraged to deliver new process insights, model development and methodological improvements. In this Special Issue, published contributions explored a range of research topics directed towards the enhanced estimation of terrestrial evaporation. Here we summarize these cutting-edge efforts and provide an overview of some of the state-of-the-art approaches for retrieving this key variable. Some perspectives on outstanding challenges, issues, and opportunities are also presented.

## 1. Introduction

The conceptually simple physics involved in the exchange of water between the surface and the atmosphere understate the critical role that this process plays in the complex dynamics driving the Earth’s carbon, water and energy cycles. As the only variable interlinking these system-level processes, the estimation of evaporation has been a focus of sustained research for many decades [1, 2]. While early process descriptions tended to focus on local scale estimation using available ground-based data, developments in satellite based remote sensing were exploited to produce some of the first large scale evaporation products [3–5]. In more recent times, efforts describing terrestrial exchanges have culminated in multi-decadal and multi-resolution products that combine satellite and numerical weather prediction model output to span local, regional and global scales [6–10].

While the various methodological approaches for flux estimation are relatively well developed [8, 11, 12], recent advances in Earth observation technologies [13], coupled with the exploitation of new retrieval and sensing techniques [14], provide an opportunity for further insights into the evaporative process. We are in a “golden-age” of Earth-observation [13], with a wide variety of distributed sensing approaches and techniques available for routine Earth system characterization, providing data at unprecedented spatial and temporal detail. New types of satellite observations, such as solar induced chlorophyll fluorescence (SIF) [15], together with advances in spatial and temporal resolution from CubeSats [16] or unmanned aerial vehicles [17], provide an opportunity to challenge both our process understanding and the models that we use to describe evaporative dynamics.

Indeed, a major driver of evolution in model descriptions is through confrontation with new data-sets. However, improving our process understanding and representation is not purely a function of increasing the resolution of observations. For instance, forcing inappropriate modeling constructs with finer and finer spatio-temporal observations may not necessarily deliver new or deeper process insights. It is important that community progress in advancing process understanding through new modeling approaches or improved physical descriptions, keeps pace with our capacity to observe the system. That is, model development efforts should aim to be transformational rather than iterative. Of course, a plateauing of model improvements is not necessarily problematic, especially if accurate results can be maintained when confronted by new observations. However, it would be naive to believe that the field of evaporation modeling (or observation) has reached its nadir, and that there are no further improvements to be made.

As a consequence, there remain many opportunities for advancing our description, estimation and understanding of the terrestrial evaporation process. In a recent contribution, Fisher et al. [14] provided a motivational call to the community to address some of the science and application questions related to evaporation estimation. These included improvements in the accuracy of retrievals, spatio-temporal resolution, multi-scale coverage and long-term monitoring, amongst many others. In light of such outstanding modeling and observational “challenges”, this Special Issue sought to explore some of the technological and methodological advances that take the first steps towards developing new paradigms in evaporation estimation. The collection of contributions spans the exploration of both new and existing techniques, high-resolution (in space and time) approaches, as well as fundamental improvements in modeling descriptions and frameworks. The following sections provide an overview of the twelve manuscripts that comprise this Special Issue, together with some concluding thoughts and perspectives.

## 2. Overview of Contributions

### 2.1 Innovative Techniques in Monitoring Evaporation

Several articles in this Special Issue explored the potential of innovative techniques to map terrestrial evaporation, from local to global scales. Pagan et al. [18] investigate the use of satellite observations of solar-induced chlorophyll fluorescence to diagnose transpiration using data from the Global Ozone Monitoring Experiment 2 (GOME-2) and a variety of FLUXNET eddy-covariance sites. Results demonstrate that SIF data normalized by photosynthetic active radiation (PAR) is empirically related to the ratio of transpiration to potential evaporation. Overall, SIF/PAR dynamics capture the effect of phenological changes and environmental stress on transpiration, adequately reflecting the timing of this variability, especially during the growing season. Moreover, the skill of state-of-the-art land surface models (LSMs) is contrasted against eddy-covariance data and SIF/PAR retrievals, indicating deficiencies in the way models estimate transpiration and suggesting that SIF data can be used to constrain the formulations of transpiration via data assimilation.

Another innovative technique is presented by Vanella et al. [19], who focused on the estimation of evaporation during water stress and irrigated conditions using local meteorology and Sentinel 2 vegetation data. The authors adjust a dual crop coefficient FAO-56 approach using electrical resistivity tomography (ERT) data to monitor the soil wetting distribution patterns during and after irrigation phases. More precisely, ERT is used to accurately estimate the wet exposed fraction and therefore the water evaporated from the soil surface. Results indicate that differences in evaporation estimates relative to in situ eddy-covariance measurements can be substantially reduced when using ERT. Moreover, the authors propose to empirically derive water stress factors by combining the eddy covariance data and the ERT-adjusted FAO-56 evaporation estimates.

The study by Cheng and Kustas [20] applies the two-source energy balance (TSEB) model to thermal observations taken from an aircraft at a few meters resolution. Results are compared to analogous evaporation estimates based on coarser scale (~90 m) data from the Advanced Spaceborne Thermal Emission and Reflection Radiometer (ASTER), and validated against data from a series of eddy covariance towers. The evaporation data using aircraft observations shows satisfactory patterns and a higher accuracy than the ASTER-based estimates when both are compared against flux tower measurements. In particular, the authors show that the high-resolution aircraft driven evaporation product is more suitable for heterogeneous terrains covering urban, agriculture, and natural vegetation, and suggest that TSEB can be used to accurately monitor the impact of urbanization on the surface energy balance using high resolution thermal observations.

### 2.2 XXX

Cheng and Kustas [20] is a clear example of the transition towards higher resolutions in evaporation remote sensing research that has been highlighted in this special issue. In another study…

#### High Resolution Remote Sensing-CubeSats/GLEAM/Thermal Revisit

Guillevic et al. (2019) [21] Impact of revisit time on ET and uncertainty

One of the critical outstanding questions in satellite remote sensing of ET is: what temporal resolution is required? There are trade-offs between temporal resolution, spatial resolution, and coverage from an engineering standpoint. Are the trade-offs linear, or are there inflection points whereby optimal compromises can be made? Guillevic et al. (2019) investigated this question in depth using *in situ* eddy covariance measurements from AmeriFlux sites. The authors evaluated the uncertainty in monthly averaged ET by removing data from the hourly/daily measurements. They found non-linearities where an optimal compromise was a 4-day revisit scenario, which provided a significant improvement in ET estimation. The 4-day scenario reduced uncertainty in ET by 40% relative to a 16-day revisit (e.g., Landsat). Moreover, they found that any return period greater than 8-days was severely detrimental to ET estimation. Interestingly, they found no significant biases based on time of day—morning or afternoon. Finally, the authors clearly point out that these estimates vary quite a lot depending on land cover type or climate zone, so their numbers should be taken contextually to the objective of the reader.

Aragon et al. (2019) [22] CubeSats

Martens et al. (GLEAM) [23]

Anderson et al. (2019) [24] demonstrates the application of a multi-scale energy balance algorithm to provide a region-specific analysis of consumptive water use associated with land use changes in the Sacramento-San Joaquin Delta region in California. The fusion approach combines ET map timeseries based on thermal observations with high spatial but low temporal resolution and with moderate resolution but frequent temporal coverage. The methodology provides an effective tool for policymakers and farmers to understand how land use conversion could impact consumptive use and demand.

### 2.3 XXX

#### Modeling/Remote Sensing/ML

Spectral Mixture Analysis (SMA) is applied by Sousa and Small (2019) [25] in a study of Landsat 8 spectral scenes over the Sacramento Valley. In this analysis they relate the radiometric surface temperature to subpixel fractions of substrate, vegetation, and dark spectral endmembers. Their proposed approach provides a physically-based conceptual framework for mapping ET that unifies two widely-used approaches by simultaneously mapping the effects of albedo and vegetation abundance on the surface temperature field. The SMA fractions are relatively insensitive to sensor spatial resolution, unlike spectral indices that currently often represent vegetation abundance in ET estimation. Because SMA uses the full reflectance spectrum it could also be integrated with planned global hyperspectral imagers.

Poon and Kinoshita (2019) [26] Remote sensing and ML

Talsma et al. (2019) [27] Sensitivity of model components

We often think of ET in terms of total ET, but it is in fact comprised of multiple sources including canopy transpiration, soil evaporation, and interception evaporation. All sources are differentially impacted by and sensitive to changes in climate and CO2. Global satellite remote sensing ET retrievals have now advanced to delineate those ET sources. Yet, no study had as yet evaluated the sensitivities of those models—PT-JPL, GLEAM, and PM-MOD—to the components until Talsma et al., (2018). The authors revealed that while each model was quite sensitive to each of the ET sources, and, naturally, different among models, that sensitivity dissipated when aggregated back up to total ET. Internally, each model had managed to balance an over-reliance on one component with an under-reliance on another component. Talsma et al. conclude that the total ET estimates, especially among PT-JPL and GLEAM, were very accurate even though the models disagreed on the contribution to total ET by the respective sources.

Moyano et al. (2019) [28] VWU in a thermal model (PT-JPL)

Multiple papers in this Special Issue noted the advancements and high accuracy of ET remote sensing algorithms, particularly for sensitive ecosystems. Arguably nowhere are water resources more constrained than in dryland systems, where people and natural ecosystems compete for limited water availability. It is these regions where accurate ET estimation may be of utmost importance to determine water use and management among sectors. Moyano et al. (2019) apply the PT-JPL ET model with dryland thermal modifications to a critical region: the World Heritage UNESCO Doñana region of Spain. They demonstrated that the remote sensing retrieval captured *in situ* measurements, giving confidence that they can use this approach for management of these critical systems.

Kumar et al. (2019) [29] investigated the level of agreement in flux partitioning among outputs from a suite of land surface models in the North American Land Data Assimilation System (NLDAS) configuration. The authors quantify the contributions of two key factors explaining inter-model disagreements to the uncertainty in total domain-wide ET: (1) contribution of the local partitioning and (2) regional distribution of ET. The results indicate that while the uncertainty in local partitioning dominates the inter-model spread in modeled soil evaporation E., the inter-model differences in T are dominated by the uncertainty in the distribution of ET over the Eastern U.S. and the local partitioning uncertainty in the Western U.S.

## 3. Concluding Thoughts and Perspectives

The monitoring and measurement of terrestrial evaporation remains a topic of considerable relevance to multi-disciplinary studies in hydrology, meteorology and ecology, as well as the agricultural and plant sciences (to name just a few). As such, it has been an area of active investigation for decades: driven in large part by the many challenging research questions that remain unanswered. These include fundamental questions on the influence and impacts that climate changes will have on our coupled water, energy and food systems; the relationship between evaporation and land-atmosphere feedbacks, extremes and other climate feedbacks [30]; to more applied aspects such as the effective management and optimization of our water resource systems (see Fisher et al. [14] for further discussion on some of these outstanding research questions).

Developments in our capacity to observe the Earth system and its dynamics provide an opportunity for further insights and improved descriptions of the terrestrial evaporative process. In recent times, the Sentinel platforms [31] have provided a needed upgrade to the series of NASA satellites that have been the backbone of Earth observation research for the last decade and more. In parallel, emerging satellite platforms [13] and technologies such as CubeSats [16] and unmanned aerial vehicles (UAVs) [17], are delivering unprecedented spatio-temporal capabilities, and facilitating novel sensor-fusion approaches relevant to enhancing evaporation [32]. Others platforms such as the ECOSTRESS sensor [33], provide a source for monitoring diurnal temperature patterns at high-resolutions, offering important insights into plant response and behavior.

To a certain degree, the theoretical constructs upon which the majority of our evaporation models are based have remained largely unchanged since the middle of the last century. As a consequence, one may start to question whether foundational concepts (for example, Penman’s “big-leaf assumption”) remain relevant in the face of observational improvements (i.e. if the capacity to observe individual leaves is routinely available). Of course, advances will not be driven solely by just increasing spatial and temporal resolutions. Certainly, one of the most important aspects required to advance the development of evaporation models is our representation of the vegetation components inherent in the partitioning between evaporation and transpiration: an aspect that will benefit not only from resolution improvements, but also new retrieval approaches [28, 29, 34]. Given the array of existing sensing systems that span the visible, near-infrared, thermal and microwave spectral ranges, efforts to utilize the varied information to better characterize land surface influences (e.g. plant form and function) are likely to provide further knowledge gains [35–37]. Likewise, new sensing approaches such as SIF [18] as well as other enhanced metrics of vegetation function and condition [32] are providing supplementary sources of data to better inform key process elements [38].

The idea of new observations spurring development and advances in physical representations is not new, but it is one that is increasingly relevant in evaporation studies. Observational improvements and model developments provide parallel (yet overlapping) pathways towards advancing our knowledge and understanding of the evaporation response, with many excellent examples comprising this Special Issue. Another aspect that is likely to become increasingly important is the use of data-driven approaches, exemplified through the adoption of artificial intelligence and machine learning techniques [39, 40]. Regardless of the path taken, it is clear that efforts towards advancing the understanding, description and interactions of terrestrial evaporation present numerous challenges and opportunities. Given the central role that this important topic plays in multi-disciplinary investigations, there is clear potential to undertake novel, innovative and impactful research towards addressing these outstanding problems.
